# A dataset of high-resolution snapshots of the viscous sublayer from direct numerical simulation of a turbulent boundary layer up to *Re*_θ_=2400

**DOI:** 10.1016/j.dib.2026.112767

**Published:** 2026-04-13

**Authors:** Joseph O’Connor, Richard D. Whalley, Andrew Wynn, Sylvain Laizet

**Affiliations:** a EPCC, University of Edinburgh, Edinburgh, UK; b School of Mechanical and Aerospace Engineering, Queen's University Belfast, Belfast, UK; c Department of Aeronautics, Imperial College London, London, UK

**Keywords:** Time-resolved snapshots, Turbulent boundary layer, Viscous sublayer, Direct numerical simulation, Incompact3d, Zarr

## Abstract

This dataset comprises time-resolved 3D fluid field data (pressure and the three velocity components) from the viscous sublayer of a canonical zero-pressure-gradient turbulent boundary layer. In total it contains 16,384 snapshots, amounting to approximately 11.1 TiB of data (pre-compression). In addition to the snapshot data, the dataset also includes time-averaged turbulent statistics over the full boundary layer for the four primary quantities (pressure and velocity) together with the second-order velocity products, enabling validation and comparison with existing literature. To create the data, direct numerical simulations were performed with the high-order flow solver Incompact3d on the ARCHER2 UK national supercomputer. Following the simulation, the raw Incompact3d outputs were converted to Zarr v3 and uploaded to a remote object store, together with accompanying materials (metadata, example scripts, licence, and readme). Other than format conversion, no additional processing has been applied. The data are hosted on the Edinburgh International Data Facility (EIDF), which provides a graphical web interface via the Comprehensive Knowledge Archive Network (CKAN) interface. Given the size and structure of the dataset, programmatic access is expected to be most convenient; accordingly, the EIDF also exposes an interface compatible with a subset of the Amazon Simple Storage Service (S3) REST API. Performance-aware storage choices were made to facilitate efficient remote access. Chunk sizes were selected to optimise anticipated common access patterns. Where appropriate, sharding was applied to reduce the number of files and the load on the remote filesystem and compression has also been applied to reduce network traffic. Example Python scripts demonstrate end-to-end usage (opening the stores, plotting, unit conversion, and chunk-aware sampling for machine-learning pipelines), lowering the barrier to entry and serving as templates for custom analyses. The dataset will enable a broad range of research activities, including developing and testing turbulence theory, training and evaluating data-driven models, and validating experimental protocols and lower-fidelity computational fluid dynamics models.

Specifications Table

**Table:** 

Subject	Engineering & Materials science
Specific subject area	A high-resolution dataset of the viscous sublayer from direct numerical simulation of a turbulent boundary layer.
Type of data	Multidimensional arrays of time-resolved snapshots and time-averaged turbulent statistics (converted to Zarr v3 from raw Incompact3d output); example access/analysis scripts (Python).
Data collection	Direct numerical simulation was performed with Incompact3d on the ARCHER2 UK national supercomputer. Prior to the run, a minor code modification was implemented to enable writing snapshot data from a selected subdomain. During execution, the simulation was monitored for statistical convergence; once convergence criteria were met, snapshots were written to disk at regular time intervals. Following completion, the raw Incompact3d outputs were converted to Zarr v3 and uploaded to the Edinburgh International Data Facility, together with accompanying material (e.g. metadata, example scripts, licence, and readme). Other than format conversion no other processing was applied to the data.
Data source location	Edinburgh International Data Facility, EPCC, University of Edinburgh
Data accessibility	**Repository name:** High-resolution snapshots of the viscous sublayer from direct numerical simulation of a turbulent boundary layer up to $Re_{\theta }=2400$ **Data identification number:**https://doi.org/10.7488/9644bb83–261d-4bd9–96b2-cddec8c9285a **Direct URL to data:** Comprehensive Knowledge Archive Network (CKAN): https://catalogue.eidf.ac.uk/dataset/eidf198-high-resolution-snapshots-of-the-viscous-sublayer-from-direct-numerical-simulation-of-a-turb Amazon Simple Storage Service (S3) REST API: https://s3.eidf.ac.uk/eidf198-highres-snapshots-sublayer-dns-tbl-re2400 **Instructions for accessing the data:** Anonymous (read-only) access is through either the CKAN interface or the S3-compatible API. Due to the size and structure of the dataset, programmatic access is recommended via a third-party Zarr-backed array-manipulation library (e.g. Xarray, Zarr-Python, Dask). New users are encouraged to begin by downloading the readme and examples via the CKAN interface or the S3 API.
Related research article	None

## 1 Value of the Data

• **High-resolution fluid field data:** The dataset provides time-resolved 3D fluid fields (pressure and the three velocity components) of the viscous sublayer from direct numerical simulation of a canonical zero-pressure-gradient turbulent boundary layer, at high temporal resolution and extent.
• **Time-averaged turbulent statistics:** To support validation and comparison with existing literature, the dataset also includes time-averaged statistics of the four primary quantities (pressure and velocity) together with the second-order velocity products.
• **Storage:** The dataset is hosted on the Edinburgh International Data Facility, operated by EPCC (University of Edinburgh). It is accessible either through a graphical web interface via the Comprehensive Knowledge Archive Network (CKAN) or programmatically via an endpoint compatible with a subset of the Amazon Simple Storage Service (S3) REST API.
• **Format:** The dataset is stored in Zarr v3, a format designed for large N-dimensional arrays on remote object stores. Zarr supports chunking for efficient random access, transparent compression to reduce network traffic, hierarchical grouping, and parallel input/output. Chunk shapes have been selected to optimise anticipated access patterns. Programmatic access is available via the S3 endpoint using Zarr-backed array-manipulation libraries (e.g. Xarray, Zarr-Python, Dask), which expose familiar array-like interfaces (e.g. slicing, arithmetic).
• **Example scripts:** Python examples are provided to demonstrate end-to-end usage, including opening the remote store, plotting quantities of interest, converting to inner (viscous) units, and implementing chunk-aware sampling for machine-learning pipelines. These examples lower the barrier to entry and serve as templates for custom analyses.
• **Use cases:** The dataset enables a range of research activities, including developing and testing turbulence theory, training and evaluating data-driven models, and validating experimental protocols and lower-fidelity computational fluid dynamics models.

## 2 Background

**Fig. 1:**
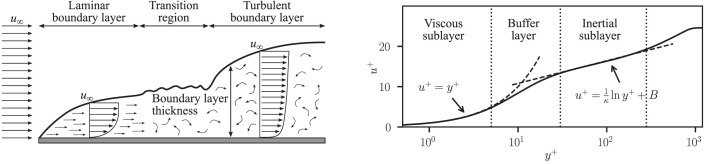
Laminar-to-turbulent boundary layer transition (left; adapted from [3], CC BY 4.0) and inner-scaled mean velocity profile across the boundary layer at $Re_{\theta }=2487$, from the present dataset (right). Dashed lines indicate the linear and logarithmic (κ=0.41,B=5.2) regions.

Turbulence remains one of the great unsolved problems in physics. Wall-bounded turbulence, in particular, governs the behaviour of many natural and engineering systems, from the drag on aircraft to blood flow in arteries [1]. However, turbulent flows are notoriously difficult to study, owing to their nonlinear, high-dimensional, chaotic, and multiscale nature. Experimental investigations are constrained by limited resolution (often pointwise), restricted sensor access, and probe intrusiveness. Computational modelling approaches also face severe challenges, including stringent resolution requirements to ensure accurate solutions and large data volumes that are challenging to store and process. Recent advances in large-scale storage infrastructure and the supporting software ecosystem now make it feasible to curate, access, and process multi-terabyte datasets, enabling public dissemination of direct numerical simulation data at unprecedented resolution and extent. With this in mind, the motivation for this work is to provide a carefully curated high-resolution dataset of the viscous sublayer [2] (see Fig. 1) within a canonical zero-pressure gradient turbulent boundary layer in a form that is conveniently accessible despite its size, with the ultimate aim of supporting new methodological developments in turbulence research, as well as providing a reference for benchmarking and validation.

Several public direct numerical simulation resources already exist for canonical wall turbulence. Representative statistics-first databases include the UT Austin channel flow database [4] and the KTH zero-pressure-gradient turbulent-boundary-layer database [5], both of which are widely used for benchmarking mean profiles, Reynolds stresses, and related statistics. For instantaneous three-dimensional fields, the Johns Hopkins Turbulence Databases provide a range of turbulent-flow datasets accessible via convenient web services [6], although their boundary layer offering is transitional rather than fully developed. For fully developed turbulent boundary layers, the Michigan Deep Blue database provides large time-resolved 3D velocity fields [7], although these are distributed as large HDF5 files that must be downloaded locally. The present dataset complements these resources by providing time-resolved three-dimensional near-wall (viscous sublayer) snapshot fields at high temporal resolution and extent, together with accompanying statistics, in a cloud-optimised Zarr layout designed for efficient programmatic access and supporting partial and random reads.

## 3 Data Description

This section provides the information needed for users to interpret and work with the dataset (see [8] for the official dataset record), including its structure, contents, dimensions, scaling, storage format, hosting infrastructure, and access details. Further details of the numerical setup and implementation, including the boundary conditions, tripping strategy, numerical parameters, and validation, are provided in the *Experimental Design, Materials and Methods* section.

**Fig. 2:**
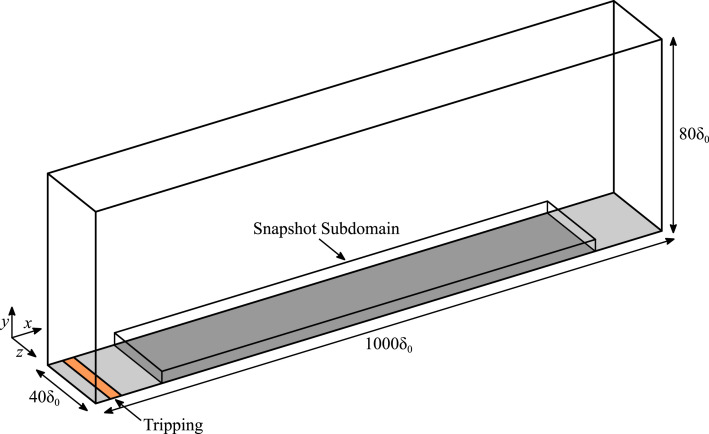
Schematic of the computational domain and snapshot subdomain.

The dataset comprises time-resolved fluid field data (velocity and pressure) from the viscous sublayer of a canonical zero-pressure gradient turbulent boundary layer, obtained via direct numerical simulation using Incompact3d [9,10]. Fig. 2 distinguishes the full computational domain from the near-wall snapshot subdomain (which spans the viscous sublayer). For the time-resolved fluid field data, only the snapshot subdomain is stored in the repository. The streamwise, wall-normal, and spanwise directions are denoted by x, y, and z, respectively. Equivalently, u, v, and w denote the instantaneous velocity components in the three spatial directions. All quantities are non-dimensionalised by the boundary layer height ($\delta_{0}$) and freestream velocity at the inlet ($u_{\infty }$) (outer scaling). Consequently, $\delta_{0}=1$ and $u_{\infty }=1$ by construction, and all fields and coordinates in the repository are provided in this non-dimensional form. If desired, users may assign physical units by selecting dimensional reference scales and rescaling accordingly. This manuscript adopts the same normalisation. However, where appropriate, inner units (denoted by the plus superscript) are also provided for reference.

The full domain dimensions are $L_{x}\times L_{y}\times L_{z}=1000\times 80\times 40$ (in units of $\delta_{0}$, as described above), with periodic conditions imposed in the spanwise direction. The time-resolved snapshots stored in this repository correspond to the near-wall snapshot subdomain (also in units of $\delta_{0}$) spanning 50≤x≤900, 0≤y≤0.1993, and 0≤z≤40. This corresponds to a momentum Reynolds number of $425\leq Re_{\theta }=u_{\infty }\theta /\nu \leq 2487$, where θ is the momentum thickness and ν is the kinematic viscosity. In the wall-normal direction the subdomain extends up to $y_{s}^{+}=13.17$ at the start of the snapshot region (x=50) and $y_{e}^{+}=10.14$ at the end of the snapshot region (x=900), where subscripts s and e denote the streamwise start and end locations of the subdomain, respectively. The wall-normal extent of the subdomain was chosen to ensure that the viscous sublayer ($y^{+}\leq 10$) is fully captured within the snapshot subdomain.

The mesh size of the full computational domain is $N_{x}\times N_{y}\times N_{z}=4097\times 513\times 256$, with uniform spacing in the streamwise and spanwise directions and non-uniform spacing in the wall-normal direction to adequately resolve near-wall effects. For the snapshot subdomain, the mesh size is $n_{x}\times n_{y}\times n_{z}=3482\times 26\times 256$. The snapshot data contains $n_{t}=16,384$ time instances of 3D fluid field data for four variables (pressure and the three velocity components), written at intervals of Δt=0.1536 ($\Delta t_{s}^{+}=0.54$ and $\Delta t_{e}^{+}=0.32$) covering a total time of ΔT=2516 ($\Delta T_{s}^{+}=8787$ and $\Delta T_{e}^{+}=5213$). In total, the snapshot data consists of four data arrays of shape 16384×3482×26×256 in double precision (11.1 TiB).

To aid validation and comparison against benchmarks in the literature (see Figs. 5 and 6 in the next section), time-averaged turbulent statistics over the full computational domain are also provided alongside the time-resolved snapshot data. In addition to the four primary variables (p, u, v, w), the statistics also include second-order velocity products ($u^{2}$, $v^{2}$, $w^{2}$, uv, uw, vw), for a total of ten data arrays with shape 4097×513×256 in double precision (40.1 GiB).

The dataset is hosted on the Edinburgh International Data Facility (EIDF), a set of computational, data-management, and secure storage services supported by the Data Driven Innovation Programme of the Edinburgh and South-East Scotland City Region Deal. As part of its offering, the EIDF provides a data publishing service via an object store with a Comprehensive Knowledge Archive Network (CKAN) interface. Given the size and structure of the present dataset, it is anticipated that users will typically access it programmatically. To this end, the EIDF also exposes an interface compatible with a subset of the Amazon Simple Storage Service (S3) REST API. Note that the object store is operated by the EIDF (not Amazon Web Services); the S3 interface is provided solely for API compatibility with existing tools.

The dataset is stored as a collection of N-dimensional arrays in Zarr (v3) format within an S3 bucket on the EIDF. Zarr is a storage specification designed for large N-dimensional arrays and enables efficient input/output on remote object stores. Key features include chunking for efficient random access, parallel processing, hierarchical grouping, and compression. The present dataset leverages these features by storing the time-resolved snapshots and time-averaged statistics in two separate groups with optimised chunk shapes. Other than converting the raw (binary) Incompact3d outputs to Zarr, no further processing has been applied to the original data.

Optimising chunk shapes for anticipated access patterns is essential for performance on remote storage. A balance is required between smaller chunks (reducing wasted network transfer) and larger chunks (reducing the number of requests), as well as filesystem considerations. For the snapshots, three access patterns are expected: (1) all spatial locations at a single timestep; (2) all timesteps at a single spatial location; and (3) all timesteps and wall-normal and spanwise locations at a single streamwise location (Reynolds number). General Zarr guidance also suggests chunk sizes of 10–100 MiB for optimal network performance [11,12]. Based on these considerations, a chunk shape of 38×8×26×256 was selected, resulting in 432×436×1×1 chunks along each dimension, with an uncompressed chunk size of 15.4 MiB. With this choice, access patterns (2) and (3) each require 432 chunks to be read from remote storage, while access pattern (1) requires 436 chunks. For the statistics data, it is anticipated that most users will load the full spanwise extent (e.g. for spanwise averaging), owing to homogeneity. Therefore, a chunk shape of 293×37×256 was adopted, giving 14×14×1 chunks and a chunk size of 21.2 MiB (uncompressed).

The snapshot chunking results in 188,352 chunks per array. With four arrays, this would amount to 753,408 files, which places a heavy load on the filesystem. To mitigate this, Zarr (v3) supports sharding, whereby multiple chunks are stored within a single file. Individual chunks are then retrieved via partial reads of the shard, at the cost of an additional metadata request (to get the offset into the file). Here, each shard contains four chunks along the two chunked dimensions (t and x), producing a shard shape of 152×32×26×256 and 108×109×1×1 shards, resulting in 11,772 files per data array (47,088 files across four arrays). For the statistics arrays, the 196 chunks per array results in 1960 files across the ten data arrays. This is negligible compared to the snapshot data. Therefore, no sharding is applied to the statistics data. While sharding is important for remote filesystem performance, it is typically transparent to end users. In contrast, understanding the chunk shape and designing access patterns accordingly is critical for good performance.

To minimise network traffic, Zarr supports a range of per-chunk compression schemes. Based on exploratory testing, lossless LZ4 (level 3) with bit shuffling was selected as it offered a satisfactory balance between compression ratio and decompression rate. Overall, the compression ratio is approximately 1.2. Again, this is typically transparent to the user.

Coordinate data for each array – (t, x, y, z) for the snapshots and (x, y, z) for the statistics – are stored alongside the data arrays and linked using array metadata attributes to ensure compatibility with popular array-manipulation libraries (e.g. Xarray). Since the coordinate arrays are 1D and negligible in size compared to the data arrays, no chunking/sharding or compression is applied. Table 1 provides a summary of the Zarr-formatted store and sub-groups.

**Table 1:** Summary of snapshot and statistics groups in the Zarr store.

	Snapshots	Statistics
Data Arrays	p,u,v,w	p, u, v, w, $u^{2}$, $v^{2}$, $w^{2}$, uv, uw, vw
Coordinate Arrays	t,x,y,z	x,y,z
Array Shape	16384×3482×26×256	4097×513×256
Array Size (GiB)	2829.1	4.0
Group Size (GiB)	11,316.5	40.1
Chunk Shape	38×8×26×256	293×37×256
Shard Shape	152×32×26×256	–
Chunk Size (MiB)	15.4	21.2
Shard Size (MiB)	247.0	–
Number of Chunks	432×436×1×1	14×14×1
Number of Shards	108×109×1×1	–
Array File Number	11,772	196
Group File Number	47,088	1960

**Fig. 3:**
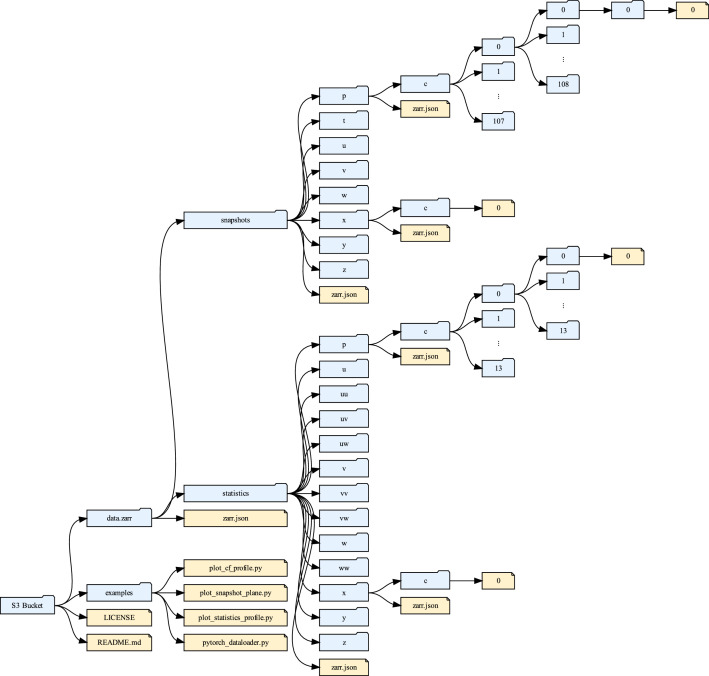
Structure and contents of the remote data repository.

Fig. 3 illustrates the structure of the remote object store. The root level is the S3 bucket endpoint (https://s3.eidf.ac.uk/eidf198-highres-snapshots-sublayer-dns-tbl-re2400). *data.zarr* contains the Zarr-formatted dataset. In practice, it is anticipated that the most convenient access will be via the S3 endpoint using third-party Zarr-backed array-manipulation libraries (e.g. Xarray, Zarr-Python, Dask), which provide array-like interfaces (e.g. slicing and arithmetic). Consequently, detailed knowledge of the on-disk layout of *data.zarr* is generally unnecessary. However, for reference, a brief description is included here. The *snapshots* and *statistics* directories are separate Zarr groups containing the respective data, and *zarr.json* is the root-level metadata file. This metadata has been consolidated so that it contains all sub-level metadata associated with each group/array, thereby reducing network requests since sub-level metadata files do not need to be fetched.

Each Zarr group contains the data arrays and associated coordinate arrays, along with its own metadata file. Likewise, each array contains its data and metadata. For clarity, Fig. 3 shows only the structure of a single data array and a single coordinate array for each group. Each sub-level within an array corresponds to the shard/chunk indices along the relevant dimensions. The snapshot arrays have four sub-levels (four dimensions), whereas the statistics arrays have three. The numbering reflects the files stored on disk: for the snapshots, indices correspond to shards whereas for the statistics, indices correspond to chunks (no sharding applied). Coordinate arrays in both groups shave a single sub-level/file because they are 1D and unchunked.

**Fig. 4:**
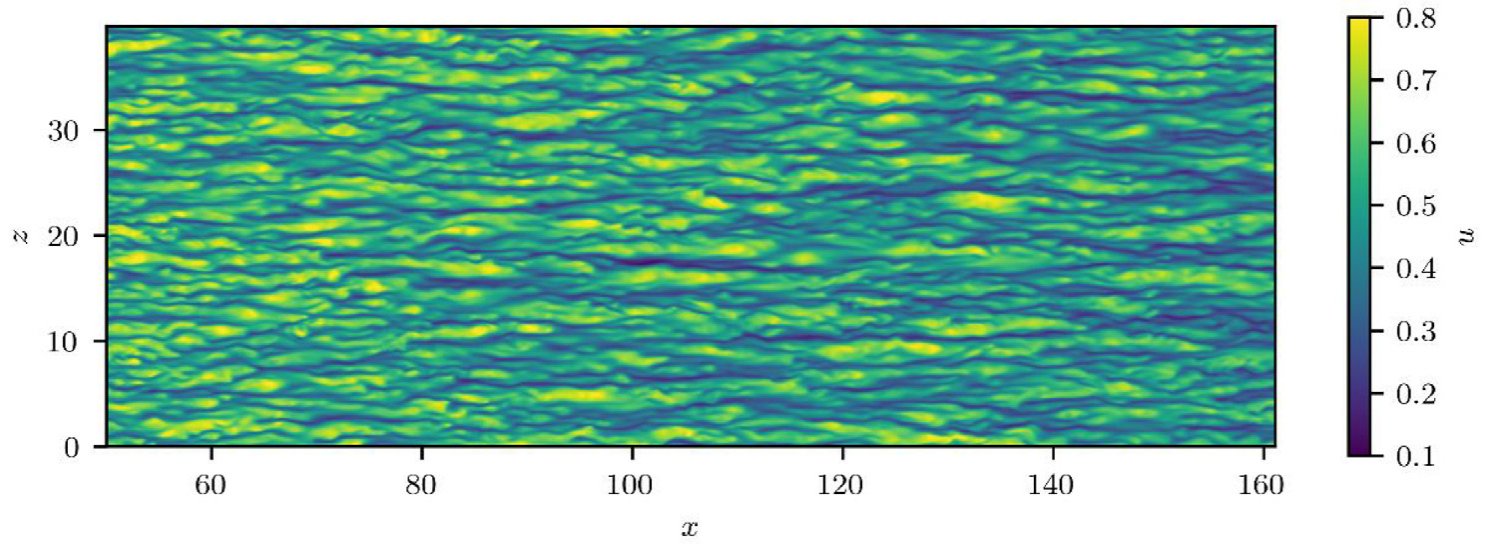
Streamwise velocity contours in the x−z plane at y=0.1993 for the first timestep.

In addition to the Zarr-formatted dataset, the S3 bucket includes a license file (CC BY 4.0), a readme, and an examples folder demonstrating access and use. The four example scripts include: *plot_cf_profile.py* (for accessing time-averaged statistics and plotting the skin-friction profile); *plot_snapshot_plane.py* (for accessing time-resolved snapshots and plotting streamwise-velocity contours); *plot_statistics_profile.py* (for accessing time-averaged statistics and converting to inner units); and *pytorch_dataloader.py* (a PyTorch DataLoader with efficient chunked sampling for the snapshot data). Fig. 4 provides a visual check for *plot_snapshot_plane.py*, which reproduces the streamwise velocity contours in the x−z plane at y=0.1993 for the first timestep. New users are encouraged to begin by downloading the readme and examples via the CKAN interface or the S3 API.

## 4 Experimental Design, Materials and Methods

The numerical simulations were performed with the high-order compact finite-difference flow solver Incompact3d [9], which is part of the open-source framework of flow solvers Xcompact3d [10]. Incompact3d is a well-established, high-performance code for scale-resolving simulations of turbulent flows and has been extensively validated across a wide range of configurations [[13], [14], [15]]. For a comprehensive description of Incompact3d the reader is referred to the literature. However, for completeness, a brief overview is given here.

The governing equations are the unsteady, three-dimensional, incompressible Navier-Stokes equations, given by:

(1) $$\begin{array}{c} \nabla \cdot u=0 \end{array}$$

(2) $$\begin{array}{c} \frac{\partial u}{\partial t}+\frac{1}{2}\left[\nabla \left(u⊗u\right)+\left(u\cdot \nabla \right)u\right]=-\frac{1}{\rho }\nabla p+\nu \nabla^{2}u+F \end{array}$$

where u is the velocity vector, t is time, ρ is mass density, p is pressure, ν is the kinematic viscosity, and F represents any external forcing. The flow is an incompressible Newtonian fluid with constant properties. In this setting density is constant and may be taken as unity without loss of generality. Note that Eq. (2) is written in skew-symmetric form to reduce aliasing errors [16].

(1), (2) are discretised using sixth-order compact finite-difference stencils. Time integration is performed using an explicit third-order Adams-Bashforth scheme, combined with an implicit Crank-Nicolson scheme for the diffusive terms in the wall-normal direction. This semi-implicit treatment helps circumvent stability constraints arising from the non-uniform mesh resolution required to accurately resolve near-wall dynamics. The pressure Poisson equation (PPE), which enforces incompressibility, is solved entirely in spectral space using three-dimensional fast Fourier transforms (FFTs). A modified wavenumber formulation [17] ensures the divergence-free condition is satisfied to machine precision. To avoid spurious pressure oscillations commonly associated with fully collocated approaches [18], the pressure field is defined with a half-mesh offset relative to the velocity field.

The structured mesh topology enables a straightforward implementation of two-dimensional domain decomposition, based on pencil structures, using the Message Passing Interface (MPI) [9]. The computational domain is partitioned into multiple subdomains (pencils), each assigned to a separate MPI process. Derivatives and interpolations in the x,y, and z directions are computed within the X,Y, and Z pencils, respectively. The three-dimensional FFTs required by the PPE solver are performed as successive one-dimensional FFTs, executed along one direction at a time. Global data transpositions, necessary to switch between pencil orientations, are carried out using all-to-all MPI collective communications. This decomposition strategy enables excellent scalability on CPU-based supercomputers, with efficient parallel performance demonstrated on up to hundreds of thousands of cores [9,10].

To generate the present dataset, a laminar Blasius solution was prescribed at the inlet, with a boundary layer height of $\delta_{0}$ and freestream velocity of $u_{\infty }$. The remaining boundary conditions were a convective condition at the outlet, a homogeneous Neumann condition in the far field, and periodic conditions in the spanwise direction. Using $\delta_{0}$ and $u_{\infty }$ as reference scales ($\delta_{0}=1$ and $u_{\infty }=1$ by construction), the kinematic viscosity (v) is determined by the specified inlet Reynolds number (e.g. $Re_{\delta_{0}}=u_{\infty }\delta_{0}/\nu$, therefore $\nu =1/Re_{\delta_{0}}$). The inlet Reynolds number was chosen to be $Re_{\delta_{0}}=1250$, corresponding to a momentum Reynolds number of $Re_{\theta_{0}}=169$. To accelerate the transition to turbulence and establish a canonical zero-pressure-gradient turbulent boundary layer downstream, a random volume forcing approach [5] was applied at x=3.5 to trip the boundary layer (orange shaded region in Fig. 2). Under these conditions, the momentum Reynolds number increased downstream to approximately $Re_{\theta }=2584$ at the outlet.

The mesh resolution in viscous (inner) units was $\Delta x_{s}^{+}=16.13$, $0.53\leq \Delta y_{s}^{+}\leq 202.51$, $\Delta z_{s}^{+}=10.32$ and $\Delta x_{e}^{+}=12.42$, $0.40\leq \Delta y_{e}^{+}\leq 155.96$, $\Delta z_{e}^{+}=7.95$. Here, the reported $\Delta y^{+}$ values refer to the full wall-normal extent of the computational domain. Within the snapshot subdomain the maximum wall-normal mesh spacing was $\Delta y_{s}^{+}=0.53$ and $\Delta y_{e}^{+}=0.41$. The time step was set to δt=0.0032, corresponding to approximately $\delta t_{s}^{+}=1.12\times 10^{-2}$ and $\delta t_{e}^{+}=6.63\times 10^{-3}$.

The flow was initialised to a laminar Blasius solution throughout the entire domain and allowed to develop until t=2000 ($t_{s}^{+}=6985$ and $t_{e}^{+}=4143$), at which point the recording of the statistics began. To monitor statistical convergence, the spanwise-averaged skin-friction profile was measured along most of the streamwise domain (35≤x≤900) at each time step, and the mean squared difference between successive time steps was used as a convergence criterion before initiating the snapshot output. Snapshot recording began at t=4500, with 16,384 snapshots written every 48 timesteps (Δt=0.1536).

A minor modification was made to the official Incompact3d code to enable writing subdomains of snapshot data. The exact code used is available at https://github.com/joconnor22/Incompact3d/tree/highres_snapshots, and the input file is provided in the examples folder at *examples/High-Resolution-Snapshots*. In total the simulation took approximately 7 days and 16 h on 64 nodes (8192 cores) of the ARCHER2 UK national supercomputer [19].

**Fig. 5:**
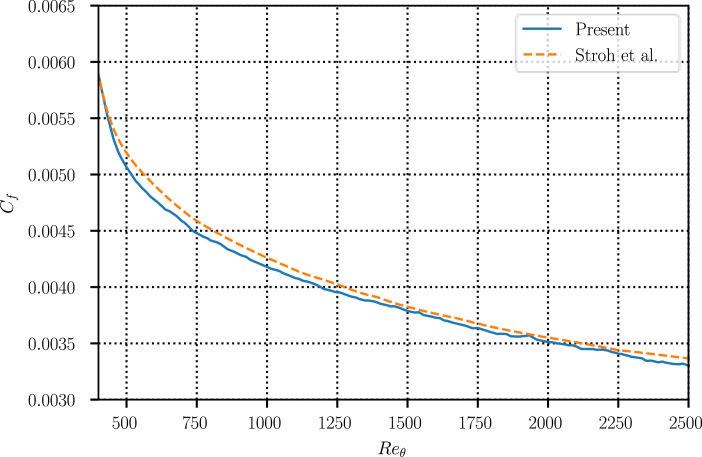
Time- and spanwise-averaged skin friction profile vs. Reynolds number.

**Fig. 6:**
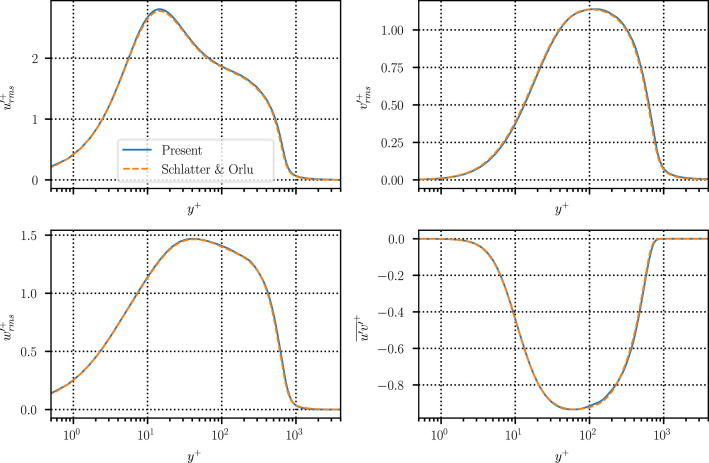
Wall-normal profiles of the time- and spanwise-averaged second-order velocity moments.

To demonstrate the validity of the present dataset, Figures 5 and 6 compare the time-averaged statistics with well-established benchmark data. Fig. 5 compares the time and spanwise-averaged skin-friction profile along the length of the domain against the results of [20], whereas Fig. 6 displays profiles of the velocity fluctuations and Reynolds stress at $Re_{\theta }=2000$ benchmarked against the data of [5]. In both cases, the agreement with the reference data is excellent, particularly for the second-order statistics shown in Fig. 6. These results confirm the accuracy of the present dataset and support its suitability for further research and analysis.

## Limitations

None.

## Ethics Statement

The authors have read and followed the ethical requirements for publication in Data in Brief and confirm that the current work does not involve human subjects, animal experiments, or any data collected from social media platforms.

## Data Availability

Edinburgh International Data FacilityHigh-resolution snapshots of the viscous sublayer from direct numerical simulation of a turbulent boundary layer up to Reθ = 2400 (Original data).
